# Medical School Clinical Trials Educational Intervention: Impact on Knowledge and Attitudes

**DOI:** 10.1007/s13187-023-02287-8

**Published:** 2023-05-11

**Authors:** Nicole E. Anzai, Jana Wieland, Richard T. Kasuya, Paula Higuchi, Kevin Cassel

**Affiliations:** 1 University of Hawai‘i John A. Burns School of Medicine, Honolulu, HI USA; 2 Office of Medical Education, University of Hawai‘i John A. Burns School of Medicine, Honolulu, HI USA; 3 University of Hawai‘i Cancer Research Center, 701 Ilalo Street, Honolulu, HI USA

**Keywords:** Medical education, Clinical trials education, Medical students, Knowledge, Attitudes, Medical school curriculum

## Abstract

Medical student knowledge and opinions of clinical research have important ramifications for how likely they will be to refer patients into clinical trials as practicing physicians. This study examined students understanding, knowledge, and attitudes about clinical trials at the start of medical school and after completion of a multi-faceted intervention designed to increase medical students’ confidence in understanding and explaining clinical trials during the pre-clinical and clinical years. Medical students were surveyed about their knowledge of and attitudes toward clinical trials in their first (*N* = 724) and third (*N* = 191) years of medical school. During the intervening years, students attend a lecture delivered by University of Hawai ‘i Cancer Center faculty, were provided a resource manual from National Cancer Institute, participated in two problem-based learning clinical scenarios, and completed an optional practicum. After completing the comprehensive clinical trials education, there were significant increases in student understanding and knowledge and a decrease in student perception that clinical trials exploit participants. Most students agreed or strongly agreed that inclusion of clinical trials in the curriculum was important and would influence their future practice. Integration of clinical trials education into the medical school curriculum improved students’ understanding of clinical research, their ability to communicate the clinical trials process, and confidence in conducting, referring to, and locating clinical trials. Medical students appreciate the importance of clinical trials in advancing medicine and medical education. Further integration of clinical trials education and opportunities to engage in research during medical school are warranted to address students’ uncertainty about the benefits of participation for patients.

## Introduction

Despite the essential role of clinical trials in the advancement of medicine, less than 5% of adult oncology patients in the USA are enrolled in clinical trials [1]. Furthermore, minority populations are disproportionately underrepresented in clinical trials [2]. Physicians play a key role in translating research to the bedside, implementing evidence-based medicine, and recruiting participants for clinical trials [3, 4]. Physician confidence that “clinical trials provide high-quality care” have been associated with higher enrollment of patients in clinical trials [5]. Considering the essential role of research to the field, it is important that physicians be equipped to understand research and feel comfortable discussing clinical trials with their patients.

Medical school curriculum is the ideal venue to foster physician understanding of clinical research and trial accessibility. The Association of American Medical Colleges reports that 62% of matriculating medical students express interest in conducting research and over 60% considered access to research experiences during medical school “important” or “very important” when selecting a medical school [6]. In addition, 78% of graduating medical students participated in research during medical school, with 51% of respondents wanting to participate in research during their career [7]. Despite high rates of research participation during medical school, there remains a gap in research knowledge with one study finding that only 25% of second- and fourth-year medical students across three universities felt adequately trained to critically assess scientific literature [8]. Medical students need clinical research education so that they will have a solid foundation from which to interpret clinical trials, be more inclined to incorporate research into their careers, and gain confidence in discussing trials with their patients.

Physicians play a key role in recruiting a representative sample of participants into clinical trials [3, 9]. Generalizability of clinical trials is drastically limited when the sample population is not representative of the general population [10]. The U.S. Census Bureau predicts that by the year 2043, the USA will be a majority-minority nation for the first time in history, yet minority groups are still largely underrepresented in clinical trials research [11]. Composed of approximately 75% ethnic minorities, the state of Hawai ‘i can contribute valuable data essential to identifying and correcting health disparities among diverse populations [12]. With 40.8% of Hawai ‘i’s physicians receiving medical training in-state [13], medical student clinical trials education at the only allopathic medical school in the state may increase the likelihood that minority populations are recruited to clinical trials and thus improve evidence-based medicine by addressing specific sub-populational healthcare needs [12]. This article discusses a unique, comprehensive clinical research curriculum intervention for medical students at a community-based allopathic medical school designed to increase knowledge and positive attitudes about clinical research based on previous findings supporting the feasibility, desirability, and importance of such an intervention [14, 15].

To our knowledge, this institution is the only medical school in the USA to require comprehensive clinical trials education as part of the curriculum. This novel educational initiative was developed in partnership with the National Cancer Institute’s Pacific region Cancer Information Service (CIS) and the University of Hawai ‘i Cancer Center’s (UHCC) Community Outreach and Education program. The goal of the program was to foster student confidence in describing the types and phases of clinical trials, identifying barriers to enrollment, explaining the importance of clinical trials to patients, and familiarizing students with resources available to patients. In addition, in 2013 the medical school introduced a year-long elective with UHCC that provides an in-depth opportunity to learn from cancer researchers, clinical trials coordinators, and community physicians about ongoing local and national research studies on a weekly basis.

## Materials and Methods

### Design

The clinical trials educational programming was based on a 2002 initiative from the NCI’s CIS Pacific Region, which served Hawai ‘i and US territories in the Pacific. The CIS established a Clinical Trial Education Coalition, which is led by the UHCC with the intent of increasing awareness of clinical trials in Hawai ‘i. The clinical trials education intervention starts with a 1-h lecture for first-year medical students taught by UHCC faculty and second-year students who have completed an immersive year in clinical research with UHCC. Content covered in the lecture includes a brief history of clinical research, basic trial design, and ethical considerations, as well as national and local study participation statistics. Each student is provided with free electronic and hard copies of the Clinical Trials Education Series (CTES) *Cancer Clinical Trials: The In-depth Program* workbook to use as a resource throughout pre-clinical and clinical years. Two problem-based learning (PBL) clinical scenarios related to oncology trials are included in the case-based learning schedule of the first pre-clinical year. Students are tasked with learning about patient access to and available resources for clinical trials as they pertain to the cases and present their findings to a small group of classmates under the supervision of a physician tutor.

Within the first month of matriculation, medical students completed the pre-intervention baseline survey composed of 22 questions based on the NCI’s Office of Education and Special Initiative-Clinical Trial Education Series for health professionals. Survey measures were anonymous and an alphanumeric code was utilized to link baseline and post-intervention measures. Participation was voluntary, signed consent was obtained from those who chose to participate, and the research project received IRB approval from the University of Hawai ‘i. Students were asked to rate their confidence in their ability to communicate clinical trials information to patients and other health professionals in the following five domains: (1) The Clinical Trials Process, (2) Clinical Trials Design, (3) Advancing Cancer Care, (4) Barriers to Clinical Trial Participation, and (5) Conducting, Referring and Locating Clinical Trials. Responses were recorded using a 5-point Likert-type scale, where 5 represented “extremely doubtful” and 1 represented “extremely confident” in their ability to communicate the information adequately. Student attitudes about clinical trials were also assessed on a 5-point Likert-type scale where 5 was “strongly disagree” and 1 was “strongly agree” with various statements about clinical trials. A detailed description of the questionnaire and preliminary data were previously described by Mitschke et al. [14].

### Participants

Baseline surveys were obtained from each cohort of first-year medical students enrolled at the medical school from 2006 through 2017 (*N* = 651). The 2012 cohort was not surveyed due to a scheduling conflict. Post-intervention surveys were obtained from third-year medical students of the 2006, 2011, and 2015 cohorts (*N* = 130) within 2 months of completing third-year clinical rotations. Scheduling difficulties prevented more consistent post-intervention administration. The post-intervention survey was identical to the baseline with the addition of three free-response questions to allow students to reflect on activities that most effectively enhanced their clinical trials education.

### Data Analysis

Data analysis was conducted using IBM Statistical Package for the Social Science (SPSS version 25). Chi-squared analyses were used to determine differences in clinical trials confidence levels and attitudes between medical student cohort years. Independent samples *t* tests were used to compare the mean pre-intervention (*N* = 651) and post-intervention (*N* = 130) knowledge and attitudes. The means of the cumulative pre- and post-intervention scores were utilized for this analysis to include the maximal number of responses given the limited number of respondents who completed both pre- and post-intervention measures. Levene’s test for equal variance was used to determine whether equal variances could be assumed and are reflected in the confidence interval reporting.

## Results

Of the 724 students who matriculated from 2006 to 2017, 651 responses were obtained for an overall baseline response rate of 90%, ranging from 78 to 97% of students in each cohort year (Table 1). A total of 191 students completed third year in the 2006, 2011, and 2015 cohorts. Post-test surveys were obtained from 130 third-year medical students for a post-intervention response rate of 68% from the cohorts surveyed. Response rates ranged from 19 to 95% (Table 1).

**Table 1:** Medical student pre-intervention and post-intervention survey response rates by matriculation year

Cohort	Baseline surveys completed	Students matriculated	Baseline survey response rate	Post-intervention surveys completed	Students graduated	Post-intervention response rate
2006	53	62	85%	58	61	95%
2007	57	62	92%	–	–	–
2008	59	62	95%	–	–	–
2009	56	62	90%	–	–	–
2010	62	64	97%	–	–	–
2011	61	66	92%	12	64	19%
2013	56	66	85%	–	–	–
2014	53	68	78%	–	–	–
2015	65	68	96%	60	66	91%
2016	63	70	90%	–	–	–
2017	66	74	89%	–	–	–

The 16 statements in Table 2 directly align with the content taught in the clinical trials curriculum. The pre-intervention survey data demonstrated that first-year medical students were uncertain at best about their ability to communicate clinical trials information adequately to patients or other health professionals with a mean confidence level ranging from 3.07 to 3.73 on a Likert-type scale where a score of 1 represented “extremely confident,” 2 represented “fairly confident,” 3 “ uncertain,” 4 “somewhat doubtful,” and a score of 5 represented “extremely doubtful.” First-year students felt the least confident in the domain of conducting, referring, and locating clinical trials. Specifically, the lowest confidence was noted in describing the types of sponsorship of cancer clinical trials (*M* = 3.73, SD = 1.213) and defining the role of the NCI in conducting clinical trials throughout the USA (*M* = 3.66, SD = 1.189). There was no statistically significant difference in medical students’ pre-intervention confidence in communicating aspects of clinical trials to patients and health providers between the cohort years. First-year medical students felt the most confident describing the purpose of randomization, stratification, and blinding in clinical trials protocols (*M* = 3.07, SD = 1.262) with no statistical improvement post-intervention.

**Table 2:** Pre-intervention and post-intervention clinical trials confidence levels

Domains	Pre-test (*N* = 651)	Post-test (*N* = 130)	Significance	Confidence interval
1. The clinical trial process				
Identify the steps in the drug development process	3.40	2.80	0.003*	0.404–0.801
Name the various types and phases of clinical trials	3.46	2.82	0.005*	0.435–0.847
2. Clinical trials design				
Review key components of clinical trial design	3.56	2.81	0.001*	0.558–0.949
Define key members of the clinical trial research team	3.61	2.89	0.001*	0.512–0.932
Describe the purpose of randomization, stratification, and blinding in clinical trial protocols	3.07	2.61	0.956	0.228–0.702
Name ways patients are monitored in clinical trials	3.46	2.75	0.076	0.485–0.923
3. Advancing cancer care				
Describe the influence of clinical trials results on the standard of cancer care	3.34	2.58	0.599	0.557–0.978
Describe clinical trials that have led to advances in cancer prevention, detection, and treatment	3.55	2.84	0.008*	0.501–0.917
Discuss the importance of professional referral and patient participation in the research process	3.32	2.65	0.858	0.450–0.880
4. Barriers to clinical trial participation				
Compare and contrast the benefits and risks of participating in clinical trials	3.34	2.71	0.634	0.424–0.849
Identify barriers that deter special populations	3.18	2.62	0.547	0.349–0.767
Recognize cost and insurance issues related to participation in clinical trials	3.52	2.79	0.008*	0.506–0.945
5. Conducting, referring, and locating clinical trials				
Describe the types of sponsorship of cancer clinical trials	3.73	3.02	< 0.001*	0.512–0.917
Define the role of the NCI in conducting clinical trials throughout the USA	3.66	2.84	0.002*	0.618–1.022
Identify methods of referring patients to clinical trials	3.61	2.82	0.043*	0.574–0.995
Demonstrate ways of locating clinical trials resources	3.52	2.84	0.002*	0.474–0.900

Ratings were based on a 5-point scale, where 1 = “I am extremely confident about my present ability to communicate this information adequately to patients and/or other health professionals” and 5 = “I am extremely doubtful about my present ability to communicate this information adequately to patients and/or other health professionals”

There is an upward trend toward increased medical student confidence in clinical trials knowledge across all the domains assessed by the post-intervention survey, summarized in Table 2. Post-intervention, the third-year medical students were significantly more confident in their overall ability to communicate clinical trials information to patients or other health professionals with a mean score ranging from 2.58 to 3.02 on the same Likert-type scale. There were significant increases in self-reported confidence in the ability to conduct, refer, and locate clinical trials, which was the area of least confidence among medical students prior to the intervention.

Seventy-one percent of respondents agreed or strongly agreed that “it is important to include information about clinical trials in the medical school curriculum.” Medical students’ attitudes toward clinical trials are summarized in Table 3. Student attitudes were on average more positive toward clinical trials, but were not significantly different from pre-intervention, with the unexpected exception that students were more uncertain about whether clinical trials are helpful, and whether subjects are treated as guinea pigs after the curriculum.

**Table 3:** Pre-intervention and post-intervention clinical trials attitudes

Attitudes	Pre-test (*N* = 651)	Post-test (*N* = 130)	Significance	Confidence interval
I feel that clinical trials are essential for the advancement of medicine	1.85	1.85	0.533	− 0.261 to 0.255
I feel that clinical trials should be considered as an option of most patients	2.30	2.11	0.387	− 0.027 to 0.402
I feel that clinical trials are useful only for patients who have tried other traditional treatments and failed†	3.03	2.86	0.659	− 0.049 to 0.380
I feel that clinical trials are not helpful and subject patients to being treated as “guinea pigs”‡	3.88	3.82	< 0.001**	− 0.204 to 0.320
I feel that it is important to include information about clinical trials in medical school curriculum	2.03	2.24	0.784	− 0.454 to 0.035
I feel that learning about clinical trials in medical school will influence my future practice	2.08	2.31	0.341	− 0.466 to − 0.002

Ratings were based on a 5-point scale, where 1 = “I agree strongly with this statement” and 5 = “I disagree strongly with this statement”

^†^The clinical trials curriculum included discussion of a wide variety of preventative, screening, diagnostic, and supportive clinical trials and aimed to decrease the misperception of clinical trials as a “last resort”

^‡^The clinical trials curriculum highlighted the protection of human subjects in clinical trials and included discussion of the purpose of the IRB, DSMB, and various safeguards built into the research process to prevent patients from harm and rectify any adverse events

In the free response section, students expressed motivation for learning about clinical trials. An example response was, “I would like more information about clinical trials because I believe that it is usually a viable option for many patients I have come across.” They felt “research is the way into the future of medicine,” and the curriculum allowed them to “appreciate more during tumor board” and that it was “helpful to know about clinical trials to discuss with attendings.” Students felt that “it has been helpful learning about new studies and findings.”

## Discussion

Post-curricular intervention, medical student knowledge regarding clinical trials improved significantly. The minimal impact of the curriculum on students’ attitudes toward clinical trials could be attributed to the inherent neutrality of the curriculum, which was designed to present a balanced view of both the benefits and drawbacks of clinical trials. First-year medical students rated themselves uncertain at best with regards to all measured aspects of communicating about clinical trials with patients and colleagues. After an integrated curriculum intervention, there were significant improvements in students’ self-reported ability to communicate the clinical trials process, and confidence in conducting, referring, and locating clinical trials. Significant increases were also seen in self-rated ability to discuss trial design, the clinical research team, describe landmark clinical trials, and discuss the costs and insurance issues regarding clinical trials participation. Improvements on these measures are especially important since an ability to facilitate discussion of clinical trials as a viable treatment option may contribute to higher accrual rates.

While attitudes were generally positive toward clinical research and its importance in the field of medicine, there was a significant increase in students’ perception that research participants are treated like “guinea pigs.” Considering that many physicians still harbor a misconception that clinical trials represent a “last resort,” it is possible that students’ perceptions were skewed by the limited population of clinical trials patients they were exposed to. A contributing factor to this perception may be the historic exclusion and exploitation of ethnic minority groups, especially indigenous persons, in research trials. The medical school studied serves the largest population of Native Hawaiian and Pacific Islanders in the USA and as such is uniquely positioned to change the narrative of historic disenfranchisement among indigenous people and ethnic minorities. Empowering diverse populations is essential to breaking the cycle of low participation leading to poor generalizability and suboptimal treatments for these minority groups. Positive exposures to research, including opportunities for students to see how research can benefit patients and improve the standard of care, may be key components of continued efforts to improve attitudes and understanding of clinical trials.

The pre-clinical and clinical years represent an important opportunity where students are motivated to learn about and engage in clinical research. Our findings indicate that medical students were receptive to the incorporation of comprehensive clinical trials education into the medical school curriculum with the vast majority agreeing that clinical trials education is important during medical school. Since few students will receive formal training in clinical research after medical school, graduating with a solid foundation in clinical trials may impact both residency performance and willingness to engage with clinical research throughout a physician’s career. Understanding the importance of clinical trials may help to ensure more precise medical care options and more confidence in referring patients to clinical trials in the long term. Thus, it is encouraging that post-intervention, students were, on average, “fairly confident” in their abilities to discuss clinical trials by their third year of medical school. These findings suggest that students may benefit from additional pre-clinical education that focuses on initiating physician–patient clinical trials communication or perhaps continued clinical trials curricula during clerkship years.

## Limitations

A limitation of the study was the low post-intervention survey response rates in the 2011 cohort which were attributed to a lack of adequate communication and opportunities to complete the survey, since the survey was emailed out instead of administered in person by the research team. Matching of within-subjects pre- and post-intervention data was limited due to incomplete post-intervention data collection as mentioned above, and discrepancies in matching the alphanumeric codes between pre- and post-surveys due to incomplete participant reporting.

## Recommendations for Future Research

Further investigation is necessary to determine the durability of this multi-faceted educational intervention using methods to increase post-intervention response rates and allow for a paired, within-subjects design. The degree to which students were involved in clinical research both before and during medical school was beyond the scope of the current project and may be controlled for in future research. Next steps include surveying medical school alumni that completed the program to assess the clinical trials knowledge retention and patient accrual rates into clinical trials in their own medical practice. Although not statistically significant, there were unexpected increases in the belief that clinical trials are only useful for patients who have failed the standard of care, and uncertainty that learning about clinical trials in medical school will influence future practice. A comparison of physicians who received a clinical trials curriculum and those who did not is needed to determine the relationship between medical school clinical trials education and practice patterns. These research findings suggest there is still work to be done to address uncertainty about which patients are appropriate candidates for research, the safety of clinical trials, and who will benefit from participation. A more formal assessment of curriculum impact might utilize a baseline and post-intervention objective structured clinical examination. Furthermore, it would be valuable to collaborate with the School of Nursing in a multidisciplinary team approach to clinical trials discussions with patients.

## Conclusion

This study demonstrated that medical students gained confidence in their clinical trials knowledge and felt better equipped to address many aspects of clinical trials after taking part in the intervention. Many medical schools have adopted the problem-based learning curriculum, generating the opportunity to similarly implement a comprehensive clinical trials education into the coursework in an effort to increase medical students’ confidence and ultimately raise clinical trial accrual rates [16]. Participation in the multifaceted clinical trials education curriculum during medical school was associated with increased student understanding and knowledge of clinical trials and medical student attitudes toward clinical trials were overall positive. This evidence-based intervention demonstrated the benefits of a comprehensive curriculum to foster greater medical student appreciation of and patient engagement in cancer clinical trials. Further integration of clinical trials education and opportunities to engage in research during medical school are warranted.

## Data Availability

Data is available on request from the authors.
